# Evolution of Sindbis Virus with a Low-Methionine-Resistant Phenotype Is Dependent Both on a Pre-Existing Mutation and on the Methionine Concentration in the Medium

**DOI:** 10.1371/journal.pone.0060504

**Published:** 2013-03-27

**Authors:** Victor Stollar, Virginia Mensah, Sandra Adams, Mei-Ling Li

**Affiliations:** 1 Department of Biochemistry and Molecular Biology, UMDNJ-Robert Wood Johnson Medical School, Piscataway, New Jersey, United States of America; 2 The Cancer Institute of New Jersey, New Brunswick, New Jersey, United States of America; 3 Department of Biology and Molecular Biology, Montclair State University, Montclair, New Jersey, United States of America; Institut Pasteur, France

## Abstract

SVlm21 is a mutant of Sindbis virus which was isolated by serial passage of virus in mosquito cells maintained in low-methionine medium; it therefore has a low-methionine-resistant (LMR) phenotype. This phenotype requires mutations at nts 319 and 321; these mutations result in Arg to Leu and Ser to Cys changes at positions 87 and 88 respectively in the viral methyl transferase, nsP1. To better understand the genesis of SVlm21, we carried out serial passages of viruses having only one of these amino acid changes, but in mosquito cells maintained in normal methionine-medium. Whether the passage was begun with SV319 or with SV321, the dominant virus population which emerged always acquired the second SVlm21 amino acid change. However, when the passage was begun with virus having neither the nt 319 or the nt321 mutation, even after many passages neither of these mutations was seen in the passaged virus population. Virus with the LMR phenotype emerged earlier when the virus encoded a wild-type RDRP (passage 4) rather than the mutant RDRP encoded by SVpzf (passage 7). When the methionine concentration in the medium of mosquito cells was increased to 250 µM, more than 20 passages were required until the LMR phenotype predominated. Competition experiments were carried out to compare the relative fitness of SVlm21, SVwt, SV319 and SV321 to each other. Our results indicated that SVlm21 was dominant to SVwt, as well as to both SV319 and SV321. However, SV319 and SV321 were able to co-exist with SVwt implying that in these mixed infection the presence of SVwt inhibited the emergence of SVlm21. Finally, our experiments highlight how a virus population by mutation and selection can adapt to the intracellular concentration of a simple metabolite, S-adenosylmethionine.

## Introduction

Changes in the properties of a virus can enhance its virulence, lead to resistance to antiviral therapy, and necessitate the modification of vaccines. Like all other replicating biological entities, viruses change and evolve by random mutation followed by selection. Indeed they provide excellent model systems for the study of these processes 1].

RNA viruses have, relative to DNA viruses, a high mutation rate, estimated to range from 10^−5^ to as high as 10^−3^ substitutions per nucleotide copied 2]. Thus, it may be expected that every time the genome of a virus such as Sindbis virus (SV) (11,703 nucleotides) (GenBank Accession number: NC_001547) is copied there would be on the average one mutation somewhere in the genome.

The high mutation rate of RNA viruses has led to the concept of “quasispecies” in describing populations of RNA viruses 2,3]. This concept implies that in any given population of an RNA virus, there is a dominant viral genotype (and phenotype) and a swarm of mutant genotypes each present in much lower numbers. Which viral genotype becomes and remains dominant in a population depends on the selective pressures exerted at any given time on the viral population. Given the high mutation rate of RNA viruses, it is likely that in any large viral population there are a small number of viral particles with a mutation at any given site in the genome. Some of these mutations would, of course, be lethal.

In the work described below, we made use of two Sindbis virus mutants isolated in our laboratory. SVlm21, is a mutant which was isolated by serial passage of SV in mosquito cells maintained in media with decreasing levels of methionine, and which has a low methionine-resistant (LMR) phenotype, i.e. it is able to replicate in mosquito cells maintained in a methionine-deficient medium 4]. Our explanation for the phenotype of SVlm21 is as follows: methionine deprivation results in a decrease in the level of S-adenosylmethionine (AdoMet); since AdoMet is a substrate for the Sindbis virus GTP methyltransferase (nsP1) 5,6], and is required for formation of the 5′ methylated G cap structures on the viral genomic and subgenomic RNAs, therefore in methionine-deprived mosquito cells, the 5′ termini of these RNAs are not capped and methylated and virus replication is inhibited. SVlm21, by making an altered GTP methyltransferase with a lower Km for AdoMet, is able to grow mosquito cells with low levels of AdoMet 7].

Two mutations are required for the LMR phenotype of SVlm21: G319U and A321U (Table 1). These mutations change Arg 87 and Ser 88 of the Sindbis virus non-structural protein, nsP1, to Leu and Cys respectively. Both mutations are needed for the LMR phenotype. The localization of these mutations was key to our identification of nsP1 as a GTP methyltransferase 6].

**Table 1:** Mutations and amino acid changes in nsP1 of SVlm21 which are required for the low methionine-resistant phenotype.

SVwt	318 CGU AGU 323	87 Arg Ser 88
SVlm21	318 CUU UGU 323	87 Leu Cys 88

The numbers in the box with the nucleotides indicate the nucleotide numbers beginning at the 5′ end of the viral genome. The numbers in the box with the amino acids indicate the amino acid positions beginning at the amino terminus of nsP1 6,20].

SVpzf, is a mutant of Sindbis virus we selected in mosquito cells on the basis of its resistance to pyrazofurin (PZF), a nucleoside analog which blocks an early step in the pyrimidine biosynthetic pathway, and thereby lowers the levels of UTP and CTP in treated cells 8]. Three mutations, all in the region encoding nsP4, the viral RNA-dependent RNA polymerase (RDRP), are required for the resistance of SVpzf to PZF, and thus for the ability to replicate in cells with low levels of UTP/CTP 8]. Since all three SVpzf mutations are in the sequence encoding nsP4, (the viral RDRP), and the resultant amino acid changes likely affect the affinity of nsP4 for at least two of its substrates, UTP and CTP, it is probable that these mutations affect the fidelity of viral RNA synthesis by SVpzf.

If we consider a Sindbis virus population as a quasispecies, it is expected that in a population of wild type virus there would be a small number of viruses with the SVlm21 mutation at nt 319 (SV319) and a small number with the SVlm21 mutation at nt 321(SV321). Under the strong selection pressure exerted by serial passage in mosquito cells maintained in a low methionine (LM) medium, it is not surprising that a dominant virus population eventually emerged with both of the SVlm21 mutations and thus the ability to replicate in methionine-deprived cells 6].

To further clarify the genesis of SVlm21, we wished to know what kind of virus population would result if we began a serial passage in mosquito cells of virus having just one of the SVlm21mutations(SV319 or SV321), but maintained the cells in normal methionine (NM) medium instead of LM medium, thus supposedly eliminating the selective pressure favoring SVlm21.

There were three possibilities. 1) The virus with only one of the SVlm21 mutations might continue as the major virus population; 2) the mutation at nt 319 or nt 321 might be lost so that the virus population would revert to the wild type; or 3) the virus might acquire the second SVlm21 mutation and the LMR phenotype. Our results indicate that the passaged virus inevitably acquired the second of the SVlm21 mutations. Furthermore, we observed that if the virus encoded the SVpzf form of nsP4 the emergence of the population with the LMR phenotype occurred several passages later than was the case with virus encoding the wt form of nsP4. We also carried out competition experiments maintaining infected cells in NM medium in order to compare the fitness of SVwt, SV319, SV321, and SVlm21 relative to each other. Our findings indicate that under these conditions SVlm21 out-competed both SVwt as well as SV319, and SV321.

## Materials and Methods

### Cells

*Aedes albopictus* mosquito cells (C7–10) were grown at 28 degrees with 5% CO_2_ in E medium (Eagle’s MEM supplemented with non-essential amino acids, and glutamine), containing 5% fetal bovine serum 9] . BSC40 cells (African green monkey kidney epithelial cells; kindly provided by Charles M. Rice and Richard Hardy [The Rockefeller University, New York and Indiana University, Bloomington, IN, respectively] 10]) were grown at 37 degrees with 5% CO_2_ in Eagle’s MEM supplemented with 10% fetal bovine serum.

### Passage of virus and titration

Beginning with the plasmid pToto, an infectious clone of Sindbis virus 11], five derivatives of pToto were generated giving a total of six different versions of pToto: 1) no mutations in nsP1 and wt nsP4, 2) no mutations in nsP1 and SV pzf nsP4, 3) nt 319 mutation in nsP1 and wt nsP4, 4) nt 319 mutation in nsp1 and SVpzf nsP4, 5) nt 321 mutation in nsP1, and wt nsP4, and 6) nt 321 mutation in nsP1 and SVpzf nsP4. Each of these plasmids was linearized by digestion with Xho I and incubated with SP6 RNA polymerase 11]. The resulting transcripts were transfected into *Aedes albopictus* mosquito cells, and after forty-eight hours at 34 degrees (Sindbis virus replicates optimally at this remperature in mosquito cells), virus was harvested. This virus was passage 0. The six passage 0 viruses were titered by plaque assay, adjusted to similar titers, and serially passaged in mosquito cells (35 mm plates) without dilution twelve times; virus was harvested at 24 hours following each passage. For each passage, mosquito cell cultures were infected with 0.5 ml of medium from the previous passage. The mosquito cells were maintained at 34 degrees after infection in 2 ml of E medium (but with 0.2% BSA in place of serum) containing a normal concentration of methionine (100 µM). Virus harvested at each passage was assayed for the ability to form plaques on a monolayer of mosquito cells maintained under an agarose overlay containing 100 µM methionine (NM), and under an overlay containing only 5 µM methionine (LM).

### Passage of Sindbis virus containing a luciferase gene

The initiating viruses for these passage series were derived from the infectious clone, pToto-luc 12] (pToto with a luciferase gene inserted into the nsP3 coding sequence; this plasmid was kindly provided by Margaret MacDonald, Rockefeller University); derivatives of pToto-luc were prepared with and without the SVpzf mutations in nsP4, and with the nsP1 mutations at nt 319 or nt 321 as indicated. Viruses were passaged as described above. To test for the LMR phenotype, mosquito cells in 24 well plates were infected with virus from each passage and maintained in NM medium or LM medium for 24 hr. Cells were lysed in 30 µl of Reporter Lysis Buffer. Fifty µl of LAR (Luciferase Assay Reagent) was added to 10 µl of cell lysate and assayed for luciferase activity using the luciferase assay system according to the manufacturer’s instructions (Promega).

### RT-PCR and nucleotide sequencing

Twenty hours after infection total RNA was extracted from infected cells using Trizol reagent (Invitrogen). One µg of total RNA and primers specific to the Sindbis virus genome (forward primer, SV25: 5′-GAATCAAACAGCCGACC; Reverse primer, W4: 5′-CTTTGGTTGCAATGCCAG) were used for RT-PCR to amplify the nsP1 coding region using the SuperScript III One-Step RT-PCR kit from Invitrogen. The cDNA was sequenced by Macrogen Sequencing Service Center using the primer SV25.

### Measurement of S-AdenosylMethionine (AdoMet) in cells

C7-10 and BSC40 cells were grown in 6 well plates until 80% confluent. Medium was removed and cells were maintained in LM, NM, or HM medium for 16h. These media contained 5 µM, 100 µM, and 250 µM methionine respectively. Cells were lysed with 200 µl of 10% trichloroacetic acid. The precipitate was removed by centrifugation for 1 min at 12,000 × g. The supernatant was back extracted with diethyl ether to a pH of 5 as described previously 13]. The concentration of AdoMet in cells was determined by the Bridge-It S-Adenosyl Methionine Fluorescence Assay following the manufacturer’s protocol (Mediomics) 14]. The fluorescent intensity was read by a FLx 800 Fluorescence Microplate Reader (BioTek).

## Results

### Serial passage of Sindbis virus in mosquito cells maintained in NM medium

Six different versions of Sindbis virus were prepared and passaged as described under Materials and Methods. To detect the LMR phenotype the titer of plaque-forming virus was measured under a low methionine (LM) overlay (5 µM methionine) and under a NM overlay (100 µM methionine). In addition to harvesting medium at each passage for determination of the virus titer, total RNA was extracted from the infected cells at each passage, and the viral RNA was sequenced over a region that included the nt 319 and nt 321 positions.

Table 2 shows the titer of total virus at each passage as measured by plaque formation under a NM overlay, and the titer of virus with the LMR phenotype as determined under an LM overlay. When the titers under the two overlays are approximately equal the dominant virus population is considered to have acquired the LMR phenotype.

**Table 2:** Passage of virus with wt or SVpzf nsP4 and one or neither of the two SVlm21 mutations in nsP1.

passage	SVwt	SVwt	SVpzf	SVpzf	SV319wt	SV319wt	SV319pzf	SV319pzf	SV321wt	SV321wt	SV321pzf	SV321pzf
	NM	LM	NM	LM	NM	LM	NM	LM	NM	LM	NM	LM
1	5.4E+05	3.0E+02	3.7E+05	4.0E+02	3.7E+05	3.0E+02	3.0E+05	6.0E+02	4.4E+05	5.0E+02	4.2E+05	5.0E+02
2	7.9E+06	3.2E+03	6.9E+06	4.8E+03	1.2E+07	2.8E+03	1.0E+07	1.7E+03	8.1E+06	5.2E+03	1.2E+07	4.7E+03
3	8.1E+06	6.9E+03	1.2E+07	3.1E+03	1.6E+07	7.2E+03	1.7E+07	2.5E+04	1.9E+07	3.1E+03	1.8E+07	6.1E+03
4	6.7E+07	5.9E+04	7.6E+07	6.4E+04	6.8E+07	7.8E+06	8.1E+07	6.3E+04	7.0E+07	9.3E+06	7.0E+07	6.1E+04
5	3.0E+09	3.4E+04	1.8E+09	4.6E+04	2.6E+09	2.6E+09	2.7E+09	5.3E+04	2.8E+09	2.1E+09	2.0E+09	7.9E+04
6	2.6E+09	3.5E+04	1.8E+09	3.5E+04	3.3E+09	2.3E+09	1.8E+09	4.9E+04	2.6E+09	2.3E+09	1.3E+09	9.0E+04
7	2.2E+09	4.1E+04	2.5E+09	3.7E+04	2.3E+09	2.3E+09	1.7E+09	1.8E+05	2.2E+09	2.8E+09	1.0E+09	1.1E+05
8	1.8E+09	4.4E+04	2.7E+09	4.5E+04	2.0E+09	2.7E+09	1.6E+09	5.0E+07	2.6E+09	2.4E+09	1.3E+09	5.0E+07
9	2.5E+09	3.5E+04	1.6E+09	4.0E+04	1.9E+09	2.4E+09	2.0E+09	5.0E+07	2.4E+09	2.7E+09	1.6E+09	4.1E+07
10	2.4E+09	5.3E+04	2.2E+09	5.3E+04	1.8E+09	2.3E+09	1.7E+09	7.5E+08	2.6E+09	2.6E+09	1.7E+09	6.6E+08
11	2.3E+09	5.2E+04	2.0E+09	2.9E+04	1.8E+09	2.3E+09	1.6E+09	1.7E+09	2.1E+09	2.3E+09	2.3E+09	2.0E+09
12	2.2E+09	7.5E+04	2.1E+09	6.1E+04	2.3E+09	2.6E+09	1.9E+09	1.9E+09	2.4E+09	2.5E+09	1.6E+09	2.0E+09

Infectious clones were constructed and virus passaged as described in Materials and Methods. Virus harvested at each passage was assayed for the ability to form plaques on a monolayer of mosquito cells maintained under agarose containing 100 µM methionine (NM), and under agarose containing only 5 µM methionine (LM). Virus populations with the LMR phenotype, i.e. with both the nt 319 and the nt 321 mutations, should give rise to approximately equal numbers of plaques under the LM overlay and under the NM overlay.

When assayed under an NM overlay (this measures the total infectious virus), the virus titers in the six different passage series gradually rose in the first four passages, and then from passages five to twelve exceeded 10^9^ pfu/ml. The titers were similar in all six passage series. In contrast, the titers of LMR virus varied not only with the passage number, but also with the genotype of the starting virus. When the starting virus encoded wt nsP4, and had the nt 319 mutation, the LMR phenotype began to emerge after four passages, and by the fifth passage the titers under the NM and LM overlays were equal, indicating that the dominant virus population had the LMR phenotype. Similar findings were observed when the starting virus encoded the wt nsP4, but had the SVlm21 mutation at nt 321.

**Figure 1:**
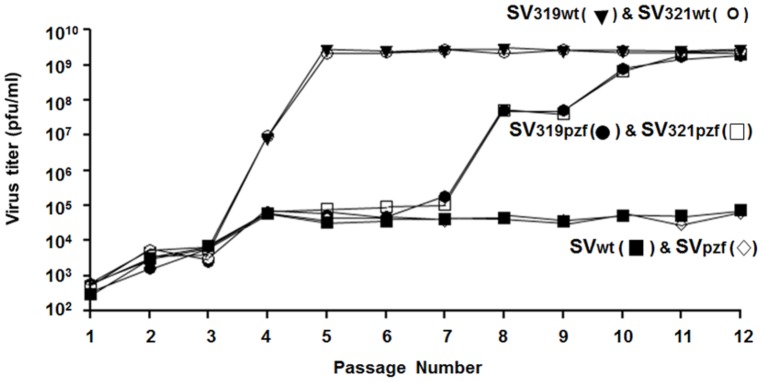
Titers of LMR virus (pfu/ml) during serial passage in mosquito cells. Medium from each passage was assayed for LMR virus by plaque formation under an LM overlay (see Table 2).

When the starting virus encoded the SVpzf form of nsP4, and had the SVlm21 mutation at either nt 319 or nt 321, a similar phenomenon was noted, but the LMR phenotype did not begin to emerge until passage 7 or 8, and virus with this phenotype did not become the dominant population until passage ten or eleven. When the starting virus had neither the nt 319 nor the nt 321 mutation, there was no emergence of virus with the LMR phenotype even after twelve passages. This was independent of whether the virus encoded the wt or the SVpzf nsP4. The emergence of the LMR virus during the passage series is depicted in Figure 1.

These passages series were repeated three times, each time beginning with passage 0 virus; similar results were obtained each time, even to the passage numbers at which the LMR virus emerged.

Sequencing of the viral RNA from infected cells indicated that the appearance of the LMR phenotype correlated with the appearance of the second SVlm21 mutation in nsP1. Thus when the starting virus had the SVlm21 mutation either at nt 319 or nt 321 and encoded the wt nsP4, the second mutation appeared first in infected cells producing passage 4 virus. However, when the starting virus had only one of the two SVlm21 mutations, but encoded the SVpzf nsP4, the second mutation was not seen until the cells were making the passage 7 virus, or three passages later than when the virus contained the wt nsP4. When virus with neither the nt 319 nor the nt 321 mutation was passaged, no mutation was seen during the twelve passages at either of these two sites. This was so whether the virus contained the wt or the SVpzf nsP4. The nt sequences were read from about position 70–75 for about 800–900 nts. Apart from the mutations at nt 319 and nt 321 no other mutations were seen. Thus there was no evidence of a general hypermutability, at least in this region of the viral genome.

To confirm these findings we repeated the experiment just described (serial passage of virus in cells maintained in NM medium), but used a different method to assay for the LMR phenotype. This was done by using viruses derived from the different versions of pToto already described, but with a luciferase gene inserted into the nsP3 coding sequence 12]. To detect the LMR phenotype with these viruses, virus from each passage was used to infect mosquito cells which were then maintained in LM medium or in NM medium. Twenty four hours later, the mosquito cells were lysed and luciferase activity measured. Virus with the LMR phenotype should grow equally well in cells maintained in LM medium and NM medium, and thus should generate roughly equal levels of luciferase activity in cells kept in the two types of media. Virus lacking the LMR phenotype should generate much less luciferase when infected cells are maintained in LM medium than when cells are maintained in NM medium.

**Figure 2:**
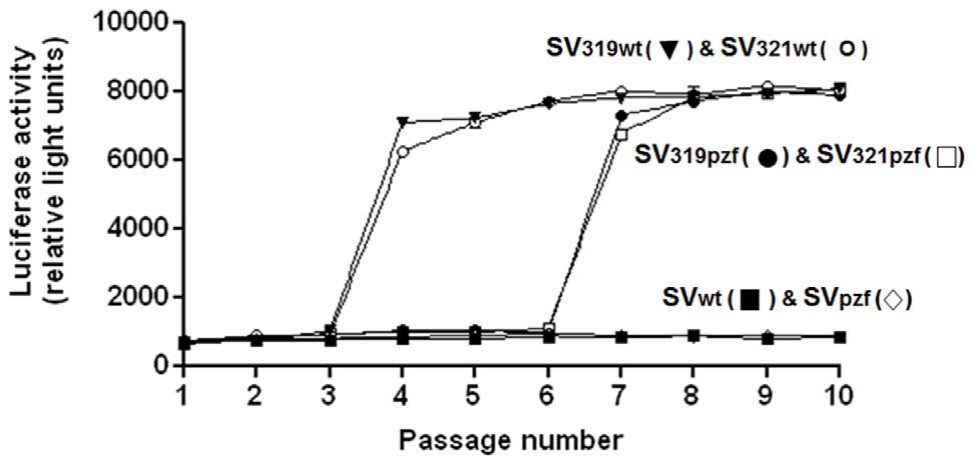
Luciferase activity of virus during serial passage in mosquito cells. The initiating viruses for the passage series were derived from the infectious clone, pToto-luc (pToto with a luciferase gene inserted into the nsP3 coding sequence), or derivatives thereof containing the SVpzf mutations in nsP4, or the nsP1 mutations at nt 319 or nt 321 as indicated. Viruses were passaged as described for Table 2. Mosquito cells were then infected with virus from each passage and maintained in NM medium or LM medium for 24 hr after which cells were lysed and assayed for luciferase activity.

Figure 2 illustrates the luciferase activity in cells infected with the passaged viruses and then maintained in LM medium. Viruses from the passage series initiated with SVluc-319-wt or SVluc-321-wt (these viruses contained the wt nsP4 and the SVlm21 mutation at nt 319 or nt 321) showed similar patterns. At passage 4, the luciferase activity increased quite abruptly and remained at this high level through the following passages. When the passage series were started with SVluc-319-pzf or SVluc-321-pzf (these viruses contained the SVpzf form of nsP4), a similar pattern was observed, but the increase in luciferase activity did not occur until passage 7. Finally when passages were begun with SVluc-wt or SVluc-pzf, viruses with neither the nt 319 nor the nt 321 mutation, there was no increase in luciferase activity over ten passages. When mosquito cells infected with the various passaged viruses were maintained in NM medium, all the values for luciferase activity were between 7,000 and 8,000 relative light units, independent of passage level (not shown), and did not show any general trend.

The results of these and the preceding experiments showed that under the conditions used, the presence of one of the two SVlm21 mutations strongly favored the emergence of the second SVlm21 mutation in the virus population, even without the selective pressure of LM medium, and that when virus encoded the wt nsP4, the second mutation emerged more quickly in the passage series than when the virus encoded the SVpzf form of nsP4.

### Passage of virus with different codons for amino acids 87 and 88 in nsP1

In the experiments just carried out, the virus with the nt 319 mutation had a CUU codon which encoded Leu 87 (Table 1). We next wished to determine whether the acquisition of the second mutation (giving rise to a Cys at position 88 of nsP1) during the passage series, and the emergence of LMR virus might be influenced by the codon encoding amino acid 87 of nsP1. Specifically, the question was whether if the passages were initiated with virus encoding a Leu at position 87 of nsP1, but with a CUG codon for Leu, instead of CUU (as in SVlm21) would the appearance of the second mutation at nt 321 (giving rise to a UGU for Cys 88) emerge as readily as it did with the virus with a CUU codon for Leu 87? Might the two U’s at nts 319 and 320 favor the appearance of another U at nt 321? We therefore carried out a passage series beginning with virus having a CUU codon for Leu 87 (as in SVlm21) and another series beginning with virus that had a CUG to encode Leu 87. In each case derivatives of pToto were prepared that encoded either the wt nsP4 or the SVpzf form of nsP4.

**Figure 3:**
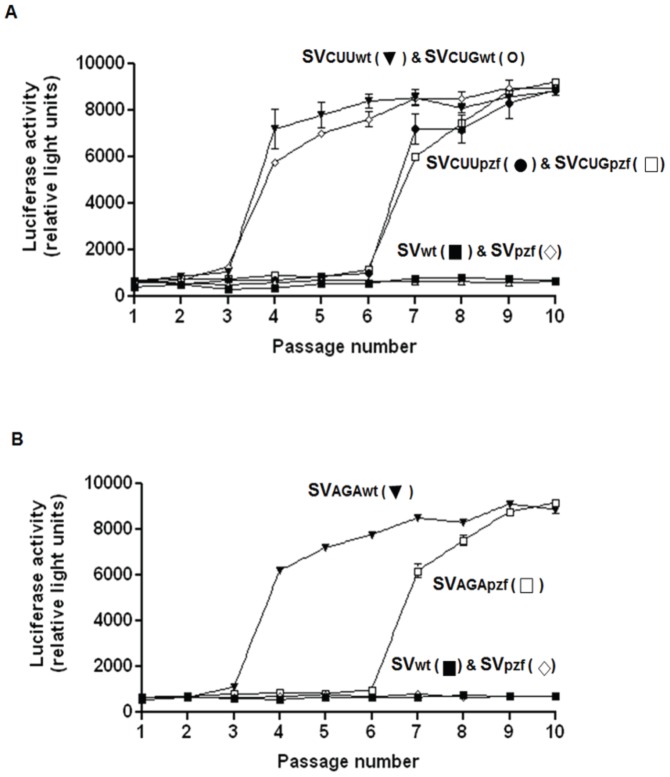
Serial passage in mosquito cells of virus with different codons for amino acids 87 and 88 of nsP1. **A:** Passage of virus with a CUG codon for Leu 87 or a CUG codon (as in SVlm21) and wt nsP4 or SVpzf nsP4. **B:** Passage of virus with an AGA codon for Arg 87 or a CGU codon (as in SVwt), and wt nsP4 or SVpzf nsP4. Viruses were constructed and passaged as described for Fig. 2.

Figure 3A shows that the appearance of the LMR phenotype, as measured by luciferase activity generated in cells maintained in LM medium, was independent of whether the starting virus had a CUU codon for Leu 87 or a CUG codon. As in the earlier experiments, the LMR virus appeared earlier when the virus encoded wt nsP4 (passage 4), compared to when virus encoded the SVpzf form of nsP4 (passage 7). Only low levels of luciferase were seen when the starting virus had neither of the two SVlm21 mutations. As in the previous experiments the appearance of the LMR phenotype correlated with the appearance of the second SVlm21 mutation, that at nt 321. It was also determined that the CUG codon for Leu 87 was maintained over the course of the ten passages.

We next asked whether the LMR phenotype would be generated so readily if two nt changes were needed to encode one of the amino acids required for the SVlm21 phenotype. Thus, a serial passage was begun as before, with virus derived from a version of pToto that encoded an Arg at position 87 of nsP1 (as in SVwt), and a Cys at position 88 (as in SVlm21); but in this case, the codon for Arg 87 was AGA, not CGU as in the wt virus (the Cys codon was UGU). This meant that two nt changes would be needed to change the Arg 87 codon to a Leu codon and give rise to the LMR phenotype.

Figure 3B shows that when virus which encoded a Cys at position 88 in nsP1, but had an AGA codon, not a CGU codon (as in SVwt) for Arg 87, virus with the LMR phenotype still became the dominant population, even though two nucleotide changes were required for the Arg to Leu change. Furthermore, the LMR virus appeared at the same passage numbers as in the previous experiments, and earlier when the virus encoded the wt SV nsP4 than when the virus encoded the SVpzf form of nsP4. As before, the appearance of the LMR phenotype correlated with the appearance of a Leu codon for position 87; but in this case the Leu codon was CUA, (not CUU as in SVlm21); this represented two changes from the AGA Arg codon.

Another very similar experiment was carried out; however, this time the virus to be passaged had a CUU codon for Leu 87 as in SVlm21, but a UCG codon for Ser 88, instead of an AGU codon as in SVwt. To generate the LMR phenotype Ser 88 would have to be changed to a Cys residue, which would require two nt changes. Viruses having the UCG or the AGU codon for Ser 88 were passaged in mosquito cells maintained in NM medium, along with control viruses having the wt codons for both amino acids 87 and 88. The appearance of the LMR phenotype was monitored as above using the luciferase assay.

The results (not shown) were very similar to those shown in Figure 3B. Independent of whether the virus had a UCG codon or a AGU codon for Ser 88, the LMR phenotype was seen at passage 4 when the virus encoded the wt nsP4, but not until passage 8 in the case of the virus that encoded the SVpzf form of nsP4. If the viruses contained the wt codons for amino acids 87 and 88, after ten passages the LMR phenotype was not seen, no matter whether the viruses encoded the wt nsP4 or the SVpzf nsP4.

Sequencing of the viral RNA made by the virus which originally had the UCG codon for Ser 88, but had acquired the LMR phenotype, revealed that the codon for Cys 88 was now UGC, not UGU as in SVlm21. This result showed that the two nt changes needed for the Ser to Cys change at position 88 of nsP1 had indeed occurred, and confirmed that even when two nt changes were needed to generate the LMR phenotype, this phenotype occurred as rapidly in the passage series as when only one nt change was required.

### Passage of virus in cells with increased levels of methionine

The preceding experiments were done with the assumption that NM medium (100 µM methionine) represented a non-selective medium. However, the consistent emergence of the LMR phenotype when the passage series were initiated with virus containing one of the SVlm21 mutations and carried out in mosquito cells maintained after infection in NM medium, led us to question this assumption. Perhaps if infected cells were maintained in medium with a higher level of methionine, presumably leading to higher levels of intracellular AdoMet, there would be much less selective pressure favoring the emergence of LMR virus which as noted above encodes a GTP methyltransferase with a higher affinity for AdoMet.

Accordingly, passage series were initiated as before with viruses containing the luciferase gene, but this time infected mosquito cells were maintained in a high-methionine (HM) medium containing 250 µM methionine for each of ten passages. In contrast to what was observed in the earlier experiments, virus with the LMR phenotype did not become the dominant population in any of the passage series. However, when the passage series was extended, LMR virus did eventually emerge, but as before only in the passages initiated with virus having either the nt 319 or the nt 321 mutation. Once again, the LMR virus appeared earlier when virus encoded the wt nsP4 rather than the SVpzf nsP4. Thus with the viruses encoding wt nsP4, the LMR phenotype was seen at passage 21, whereas with virus encoding the mutant nsP4, not until passage 24. When the passages were initiated with virus having neither the nt 319 nor the nt 321 mutation, the LMR phenotype was not seen even after 28 passages.

When we first described SVlm21 4] we observed that although the replication of Sindbis virus in mosquito cells was sensitive to methionine-deprivation, such was not the case for replication in vertebrate cells. This might be explained if the AdoMet synthetase (the substrates for which are methionine and ATP) of mammalian cells were more efficient in maintaining adequate levels of AdoMet even in the face of a low intracellular level of methionine. If such were the case, there would be little selective advantage for the LMR phenotype in virus passaged in mammalian cells maintained after infection in LM medium, let alone NM medium.

To determine whether the LMR virus would emerge in mammalian cells a passage series was carried out with the six viruses as already described for Figure 2, but this time in BSC40 cells maintained after infection in NM medium. After 10 passages there was no evidence for a dominant virus population with the LMR phenotype. The passage series was therefore continued. As was the case when viruses were passaged in mosquito cells in HM medium, the LMR phenotype did eventually emerge when the starting viruses had either the LMR mutation at nt 319 or nt 321; but as before it appeared first in the viruses with the wt nsP4 (passage 22), and only later with viruses encoding the SVpzf nsP4 (passage 26).

To compare the levels of AdoMet in C7–10 cells and BSC40 cells, we measured the concentrations of this metabolite in both cell types maintained in medium containing different concentrations of methionine. As shown in Table 3, with cells grown in NM medium, the concentration of AdoMet in BSC40 cells was about 40% higher than that in C7–10 cells. More notable are the observations that in BSC40 cells maintained in LM medium, the AdoMet concentration was still 80% of that seen with cells in NM medium; in contrast, when C7–10 cells were grown in LM medium, the AdoMet concentration was only 23% of that in cells grown in NM medium. In C7–10 cells grown in HM medium, the AdoMet concentration was similar to that seen in BSC40 cells grown in NM medium.

**Table 3:** S-adenosylmethionine concentrations in cells.

Cell type	medium	[AdoMet] pmole/µg protein
C7–10	LM	1.5 ± 0.22
	NM	6.5 ± 0.60
	HM	9.5 ± 0.49
BSC40	LM	7.3 ± 0.71
	NM	9.2 ± 0.26

C7–10 and BSC40 cells were grown for 16 hr in 6-well plates in media with different concentrations of methionine. AdoMet was extracted and measured as described under Materials and Methods. Total cellular protein in each well was determined by Bradford assay (Bio Rad). Mean values and standard errors from triplicate samples are shown.

### Competition experiments

To shed more light on the emergence of virus with the LMR phenotype, we wished to determine how the various viruses we have been studying competed with each other. The preceding experiments showed that when passaged in mosquito cells maintained in NM medium, the SVlm21 virus always out-competed both the virus with only the nt 319 mutation and the virus with only the nt 321 mutation. However, it was also important to know which virus would prove the more fit during serial passages when we co-infected cells with 1) wild type virus and SVlm21, 2) wild type virus and virus with only the nt 319 mutation, or 3) wild type virus and virus with only the nt 321 mutation. In all these experiments cells were maintained in NM medium.

These experiments were set up by co-infecting cells, with two different viruses at an MOI of one, serially passaging the viruses and then assessing the outcomes by determining the nts at positions 319 and 321.

**Figure 4:**
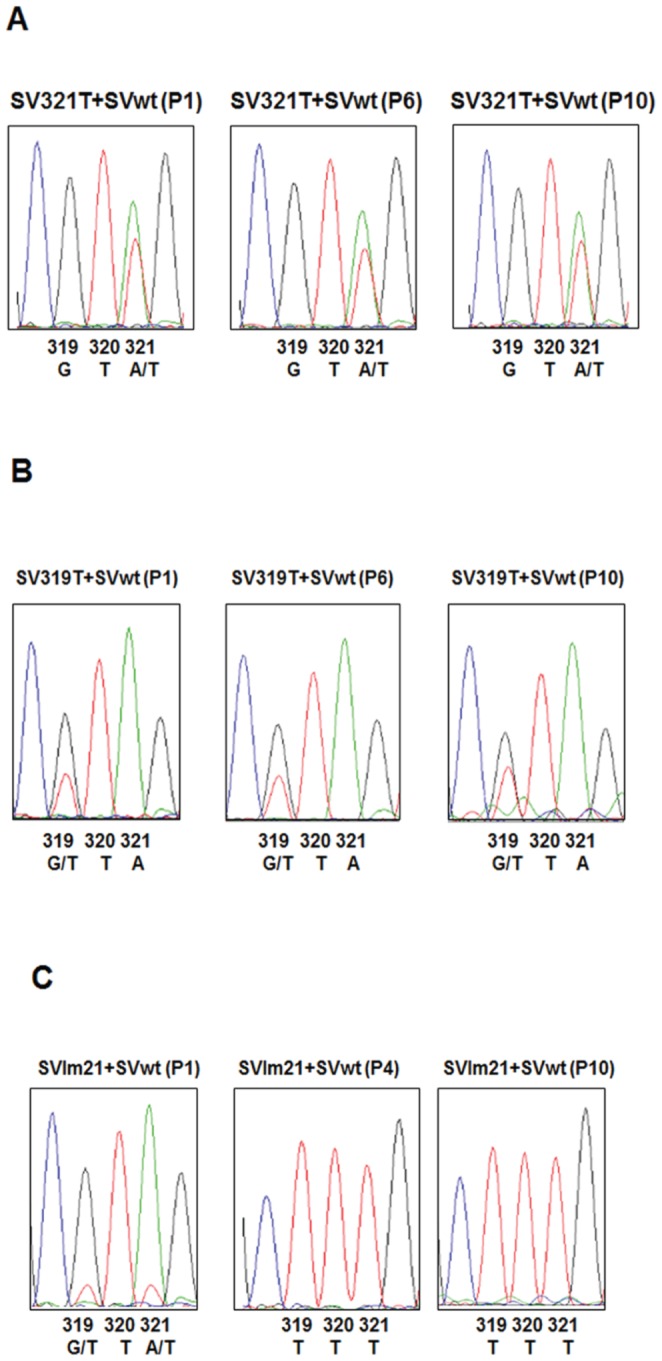
Competition experiments between SVwt, SV319, SV321, and SVlm21. C7–10 cells were co-infected with two different viruses at equal MOIs (MOI = 1) and the viruses serially passaged. Total RNA was extracted. RT-PCR was carried out using total cellular RNA and primers specific to the Sindbis virus genome as described in Materials and Methods. The nts at positions 319 and 321 were determined by sequencing. P1, P6 and P10 indicate the passage number. A: green, C: blue, G: black, and T: red. **A:** SV321T and SVwt. **B:** SV319T and SVwt. **C:** SVlm21 and SVwt.

Figure 4A illustrates the result when cells were co-infected with virus having only the mutation at nt 321 and wt virus. In passage 1, the infected cells made two species of viral RNA, one having the wild type nt, A (green), at position 321, and one with the SVlm21 nt, U (red), at this position. A similar pattern was observed with the virus population after passages 6 and 10. We conclude that both viruses were able to co-exist for ten passages under these conditions, and that neither out-competed the other, nor was the emergence of virus with both mutations observed.

A similar result was seen when cells were co-infected with virus having only the nt 319 mutation and wt virus. Figure 4B shows that at passages 1, 6, and 10 two species of viral RNA were present in the infected cells. One species representing the wt virus had a G (black) at nt 319, and the other had a U (red) representing the nt 319 mutation. We conclude that the wt virus and the virus with the nt 319 mutation were also able to co-exist at least over ten passages, and that neither out-competed the other.

A different situation was seen when cells were co-infected with wt virus and SVlm21. Figure 4C shows that cells infected with the passage 1 virus made two species of viral RNA one with the wt sequence, GUA, at nts 319–321, and one with the SVlm21 sequence, UUU. At passage 1, the wt sequence greatly predominated over the SVlm21 sequence, likely due to a greater amount of wt virus in the starting inoculum. By passage 4, however, the intracellular viral RNA contained exclusively the SVlm21 sequence, 319-UUU-321. This pattern did not change up to passage ten. Thus, SVlm21 out-competed the wt virus.

## Discussion

Most notable among our findings is that even in cells maintained in NM medium, the serial passage in mosquito cells of virus having either of the two SVlm21 mutations led to the acquisition of the second of the SVlm21 mutations and thus the LMR phenotype. This would not be surprising when virus was passaged in cells in an LM medium where there would be strong selective pressure for this phenotype. However, it was surprising that this also occurred when the serial passage was done in cells in NM medium or even in HM medium, although in the latter case the appearance of the second mutation and the LMR phenotype was much delayed.

The observation that an increased methionine concentration in the medium and the resulting increase in the intracellular concentration of AdoMet delayed the appearance of virus with the LMR phenotype is consistent with the idea that these conditions reduce the selective pressure favoring that phenotype. Conversely, reducing the AdoMet in the infected cells would increase the selective pressure favoring the LMR phenotype as was illustrated by our initial selection of SVlm21 4].

Given that the various Sindbis virus populations represent in each case a quasispecies, then even when a passage is begun with virus having only one of the SVlm21 mutations, there may already be in each stock very small numbers of virions with both of the SVlm21 mutations. What we were assessing in our experiments, however, were the properties of the dominant virus populations. The point at which a dominant virus population emerges that has both of the SVlm21 mutations is clearly dependent on the selective pressures exerted, the most important of which must surely be the concentration of AdoMet in the infected cells.

In considering the factors influencing the emergence of virus with the LMR phenotype, it was important to determine the fitness of SVwt, SVlm21, SV319, and SV321 relative to each other. The results shown in Table 2 and Figures 1 and 2 clearly indicate that in mosquito cells in NM medium, SVlm21 out-competes both SV319 and SV321_._ To compare the relative fitness of the other viruses, we carried out competition experiments as described above. Figure 4 demonstrates that SVlm21 readily out-competed SVwt. On the other hand, SVwt and SV321 were able to coexist throughout the passage series with neither out-competing the other. The same was true with respect to SVwt and SV319. Thus among these viruses, under the conditions used, SVlm21 was the dominant virus. However, it is also notable that although SVlm21 became the dominant virus when a passage was begun with either SV319 or SV321_,_ the presence of SVwt along with SV319 or SV321 appeared to inhibit the emergence of SVlm21 as the dominant virus population. Why this is so is not known. The results of the competition experiments are also consistent with the observations that when a passage was begun with either SV319 or SV321, the result was always a virus population that had acquired the second SVlm21 mutation rather than a population that had lost the first SVlm21 mutation.

In carrying out these experiments we also wished to see whether the SVpzf mutations in nsP4 would influence the emergence of a virus population with the second SVlm21 mutation. We found consistently that in every passage series, virus with the SVpzf form of nsP4 acquired the LMR phenotype several passages later than did virus with the wt form of nsP4. By itself this does not permit the conclusion that the SVpzf RDRP has a higher fidelity, i.e. is less mutagenic than the SVwt RDRP, but it does recall the observations of Pfeiffer and Kirkegaard 15] who reported that a poliovirus mutant resistant to the nucleoside analog, ribavirin, was slower to develop resistance to guanidinium than was wild type virus. Similarily, Coffey et al. 16] observed that a single mutation in the Chikungunya virus nsP4, that was associated with resistance to ribavirin and 5-fluorouracil, increased replication fidelity and reduced genetic diversity.

Our findings 1) that when a passage was begun with virus that had neither of the SVlm21 mutations, neither of these mutations appeared even after 12 passages and 2) that if a passage was begun with virus that had one of the SVlm21 mutations, then inevitably the second mutation appeared during the passage, calls to mind the paper by Blount et al., 17] describing work with *E. coli* cultured for many thousands of generations, in which the occurrence of a specific mutation making the organism able to utilize citrate appeared to be dependent on a prior mutation(s) in that population. They referred to this phenomenon as “historical contingency”.

Although much work has been done describing mutations in viral genomes that relate to resistance to antiviral drugs, little has been reported concerning how particular viruses, by mutation or selection, have adapted to the concentrations of intracellular metabolites so as to replicate optimally in the host cells that they infect. This is an especially interesting question for arthropod-borne viruses which must replicate in two different host types that are widely separated phylogenetically, and have significant differences in their biochemistry and metabolism. It is likely that the recently developing field of metabolomics will have much to teach us in this regard. For example, global profiling of changes in metabolites in Sindbis virus- infected vertebrate and insect cells will indicate how virus infection influences the metabolic pathways in both cell types and identification of key metabolites in infected cells will be useful for biomarker discovery 18] 19].
